# SENP1 promotes proliferation of clear cell renal cell carcinoma through activation of glycolysis

**DOI:** 10.18632/oncotarget.12606

**Published:** 2016-10-12

**Authors:** Baijun Dong, Yujing Gao, Xunlei Kang, Hongchang Gao, Jin Zhang, Hua Guo, Mingjian J You, Wei Xue, Jinke Cheng, Yiran Huang

**Affiliations:** ^1^ Department of Urology, School of Medicine, Renji Hospital, Shanghai Jiao Tong University, Shanghai, China; ^2^ Key Laboratory of Fertility Preservation and Maintenance of Ministry of Education, Department of Biochemistry and Molecular Biology, Ningxia Medical University, Yinchuan, Ningxia, China; ^3^ Department of Biochemistry and Molecular Cell Biology, Shanghai Jiao Tong University School of Medicine, Shanghai, China; ^4^ School of Pharmacy, Wenzhou Medical College, Wenzhou, China; ^5^ Department of Hematopathology, University of Texas MD Anderson Cancer Center, Houston, USA; ^6^ The Graduate School of Biomedical Science, University of Texas MD Anderson Cancer Center, Houston, USA; ^7^ Current address: North Shore LIJ Health System, New York, USA

**Keywords:** SENP1, glycolysis, HIF-1α, clear cell renal cell carcinoma

## Abstract

Metabolic shift toward aerobic glycolysis is a fundamental element contributing to the development and progression of clear cell renal cell carcinoma (ccRCC). We and others previously observed enhanced glycolysis and diminished tricarboxylic acid (TCA) cycle activity in ccRCC tissue. Here, by integrated gene expression and metabolomic analyses of 36 matched pairs of tumor and adjacent normal tissues, we showed that expression of Sentrin/SUMO-specific protease 1 (SENP1) is positively associated with glycolysis levels in ccRCC. Moreover, SENP1 knockdown in RCC4/VHL cells downregulated expression of key glycolytic enzymes under normoxic and hypoxic conditions and inhibited cell proliferation under hypoxic conditions, possibly due to ineffective deSUMOylation and stablization of Hif-1α related to the SENP-1 deficiency. Finally, SENP1 expression correlated positively with tumor pathological grade and was an indicator of poor overall survival and advanced tumor progression in ccRCC. Altered VHL gene function is found in 60–90% ccRCC cases of ccRCC, but therapies targeting VHL-related signaling pathways have been ineffective, spurring exploration of alternative pathological signaling events. Our results provide a possible mechanistic explanation for the role of SENP1 in the initiation and development of ccRCC with normal VHL activity, and identifies SENP1 as a potential treatment target for the disease.

## INTRODUCTION

Renal cell carcinoma (RCC) is the ninth most common cancer worldwide, with about 337,860 new cases diagnosed in 2012 [1]. Due to lack of early warning signs and symptoms, and effective treatments for patients with advanced disease, RCC has become one of the ten leading causes of cancer death for males in developed countries [2]. Clear cell renal cell carcinoma (ccRCC), the most common form of RCC, is characterized by inactivation of the Von Hippel-Lindau (VHL) tumor suppressor gene and subsequent stabilization of hypoxia-inducible factors (HIF-1α and HIF-2α), which is in turn promote angiogenesis, cell migration, and increased metabolism [3]. The loss or mutation of the VHL gene is found in 60–90% ccRCC cases, prompting the use of agents targeting circulating VEGF and VEGF receptors for ccRCC treatment [1]. However, these agents have yet to effectively improve overall patient survival. Further studies have revealed that ccRCC is a heterogeneous cancer with disparate genetic and molecular alterations beyond VHL mutation, such as mutations in genes encoding chromatin remodeling proteins, like polybromo 1(*PBRM1*) [4] and SET domain containing 2 (*SETD2*) [5], both associated with high tumor stage and poor prognosis. These studies suggest that additional genetic/epigenetic events should be considered to explain the diverse oncogenic and proliferative behavior of ccRCC.

Accumulation of epigenetic modifications has emerged as a key mechanism regulating ccRCC tumor formation [4, 6–9]. SUMOylation, covalent and reversible binding of small ubiquitin-like modifier (SUMO) to a target protein, plays an essential regulatory role in protein stability and normal function [10]. One report showed that patients with SUMOylation-defective MITF germline mutation are predisposed to melanoma and RCC through activation of HIF-1α [11]. The dynamic process of SUMOylation is catalyzed by SUMO-specific ligases and Sentrin/SUMO-specific proteases (SENPs), which conjugate SUMO polypeptides (including SUMO-1, SUMO-2, SUMO-3, and SUMO-4) to and deconjugate SUMO polypeptides from target proteins, respectively [10]. SUMO1/sentrin specific peptidase 1 (SENP1) specifically promotes the maturation of SUMO-1 to -3 by cleavage of their precursors at C-terminal residues, and removes these SUMO isoforms from modified proteins [12]. To date, many important transcription factors, transcriptional co-regulators, or chromosome-remodeling regulators such as HIF-1α, HDAC-1, and SIRT1 are reported to be SENP1 substrates, linking the function of SENP1 to normal cellular process like cell development and differentiation, mitosis, and apoptosis [13–15], and pathogenic processes such as tumorigenesis [10]. It was found that SENP1 was overexpressed in precancerous prostate intraepithelial neoplasia (PIN) and prostate cancer, and was positively correlated with the expression level of androgen receptor Overexpression of SENP1 in prostate cancer cell lines promotes cancer progression and metastasis [16–18], indicating SENP1 functions as an oncogene.

In our previous study, we found that SENP1 regulates the hypoxic response by regulating HIF-1α stability and subsequently VEGF, Glut-1, and EPO expression. The SUMOylation of HIF-1α serves as a signal for its ubiquitin-dependent degradation; SUMOylated HIF-1α binds to VHL, an E3 ligase for HIF-1α ubiquitination, and is degraded even under hypoxic conditions. Nevertheless, SENP1 could remove SUMO from SUMOylated HIF-1α to stabilize HIF-1α, allowing HIF-1α to regulate hypoxia signaling as a transcription activators [14, 19]. Genes involved in glucose metabolism, such as glycolytic enzymes and glucose transporter Glut-1, are well-known targets of HIF-1α, activation of which leads to an increased glycolytic flux and inhibited oxidative phosphorylation in ccRCC, even under aerobic conditions [20]. Previous work from our lab and others showed that all ccRCC is characterized by enhanced glycolysis to maintain homeostasis of energy metabolism and anabolic metabolism and association with tumor metastasis [21–24], but mutation of the VHL gene was only observed in 35% of our ccRCC patient group [25]. Given the regulatory role of SENP1 on HIF-1α stability, we speculate that SENP1 is a crucial regulator of HIF-1α-dependent metabolic reprogramming in the presence of VHL in ccRCC.

In the present study, we explored the role of SENP1 in regulation of glycolysis and ccRCC progression. Moreover, we evaluated the association between SENP1 expression and clinicopathological factors and analyzed its impact on overall survival and disease progression in ccRCC.

## RESULTS

### High SENP1 expression level is associated with enhanced glycolysis in ccRCC

**Figure 1:**
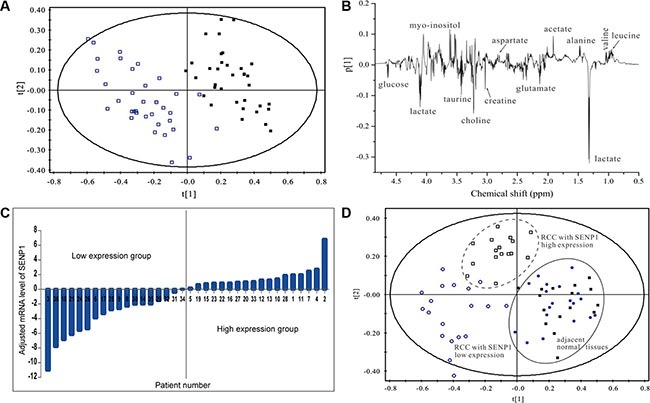
High SENP1 expression level is associated with enhanced glycolysis in ccRCC (**A**) Metabolite profiles between ccRCC tumor tissues and adjacent normal tissues. PLS-DA scores plots based on ^1^H NMR spectra of extracts obtained from tumor tissues (□) and paired normal adjacent tissues (■) of 36 ccRCC patients. (**B**) Loading plot revealing the spectral regions responsible for the discrimination of the PLS-DA model shown in (A). (**C**) Relative mRNA expression levels of SENP1 in ccRCC. Expression levels of SENP1 in the 36 pairs of human ccRCC tumor tissues (T) and normal adjacent tissues (N) were tested using qRT-PCR. The ratio of T/N mRNA level for each pair was transformed with log base 2, which represent the relative mRNA expression level of SENP1. (**D**) Metabolic profile of ccRCC was associated with the expression level of SENP1. PLS-DA scores plots based on ^1^H NMR spectra of ccRCC tumor tissues with relative SENP1 high expression (□) and SENP1 low expression (O), and the matched adjacent tissues of ccRCC with SENP1 high expression (■) and low expression (●).

To investigate the role of SENP1 in ccRCC glycolysis, we first evaluated the relationship between SENP1 expression and the level of glycolysis in ccRCC. ccRCC tumor and adjacent normal tissues were examined using ^1^H NMR-based metabonomics technology and analyzed by partial least squares discriminant analysis (PLS-DA) model, as shown in Figure 1A. A significant separation of metabolic profiles were found between tumor and normal tissues. The spectral regions responsible for the discrimination of the PLS-DA model are shown in the corresponding loading plot (Figure 1B). Compared to the adjacent normal tissues, the levels of lactate and pyruvate dramatically increased in ccRCC tumor tissue, while the concentration of malate and fumarate decreased, suggesting an enhancement of glycolysis and impaired TCA cycle in ccRCC tumor (Table 1). We quantified SENP1 mRNA in the 36 pairs of ccRCC tumors (T) and adjacent normal tissues (N), and divided the tumor samples into SENP1 high-expression and low-expression groups according to the log2 value of the ratio of T/N SENP1 mRNA level for each pair (Figure 1C). As shown in Figure 1D, the metabolic profiles of these two groups could be distinctly discriminated from each other, while the corresponding adjacent normal tissues of the two groups exhibited a similar metabolic profile. By target metabolite examination, we found that the levels of lactate and pyruvate were significantly higher in tumor from the SENP1 high-expression group than in the SENP1 low-expression group (*P* < 0.01, Table 2), suggesting a potential positive correlation between SENP expression level and glycolysis in ccRCC tumors. In addition, we noticed that the concentrations of malate and succinate, intermediate TCA cycle metabolites, were increased, which may be ascribed to previously reported SENP1 regulation of mitochondrial biogenesis [26].

**Table 1:** Summary of the relative changes of metabolite levels in extracts of tumor tissues compared to paired adjacent tissues of ccRCC patients as indicated by the PLS-DA loading plots

Metabolite	δ ^1^H	Vip	Change in ccRCC	Metabolite	δ ^1^H	Vip	Change in ccRCC
Leucine	0.96	2.23	↓	Aspartate	2.82	1.48	↓
Isoleucine	1.01	1.08	↓	Creatine	3.03	2.71	↑
Valine	1.04	1.75	↓	Choline	3.20	3.17	↓
Lactate	1.32	7.86	↑	Betaine	3.25	0.26	↑
Alanine	1.48	2.51	↓	Taurine	3.42	2.18	↑
Acetate	1.91	3.82	↓	Glycine	3.55	0.30	↓
Methionine	2.14	2.05	↓	myo-Inositol	3.53	5.02	↓
Glutamate	2.36	2.12	↑	Glucose	5.23	2.61	↑
Succine	2.40	1.25	−	Fumarate	6.52	1.07	↓
Glutamine	2.44	1.16	↑	Tyramine	7.20	1.08	↓
Pyruvate	2.47	0.59	↑	Phenylalanine	7.34	0.89	↓
Methylamine	2.63	0.73	−	Uridine	7.86	0.14	−
Malate	2.68	1.38	↓	Hypoxanthine	8.18	1.05	↓

Note: Marks indicate the direction of the change, i.e. ↓ for decrease, ↑ for increase, – for no change.

**Table 2:** Quantitative comparison of metabolites in ccRCC with high and low SENP1 expression level groups

Metabolites	SENP1 low-expression group	SENP1 high-expression group
myo-Ins	46.81 ± 4.98	66.59 ± 4.51^*^
Taurine	126.00 ± 3.72	139.60 ± 6.14^**^
PCHO	148.56 ± 11.17	144.18 ± 12.89
Creatine	156.37 ± 17.21	161.78 ± 14.82
Aspartate	60.67 ± 3.45	69.27 ± 4.96
Malate	41.08 ± 5.37	53.53 ± 5.38^*^
Pyruvate	124.24 ± 6.13	147.62 ± 9.16^**^
Succine	58.39 ± 9.12	72.67 ± 8.13^*^
Glutamate	119.16 ± 7.06	117.94 ± 4.76
Acetate	43.57 ± 5.74	44.07 ± 2.96
Alaline	87.54 ± 6.52	82.62 ± 4.56
Lactate	140.92 ± 3.78	155.25 ± 6.19^**^

* *P* < 0.05;

** *P* < 0.01.

Compared to SENP1 low expression group. The values were normalized to the metabolites concentration of the paired adjacent tissues.

### SENP1 upregulates the expression of key glycolytic enzymes and inhibits cell proliferation in ccRCC

**Figure 2:**
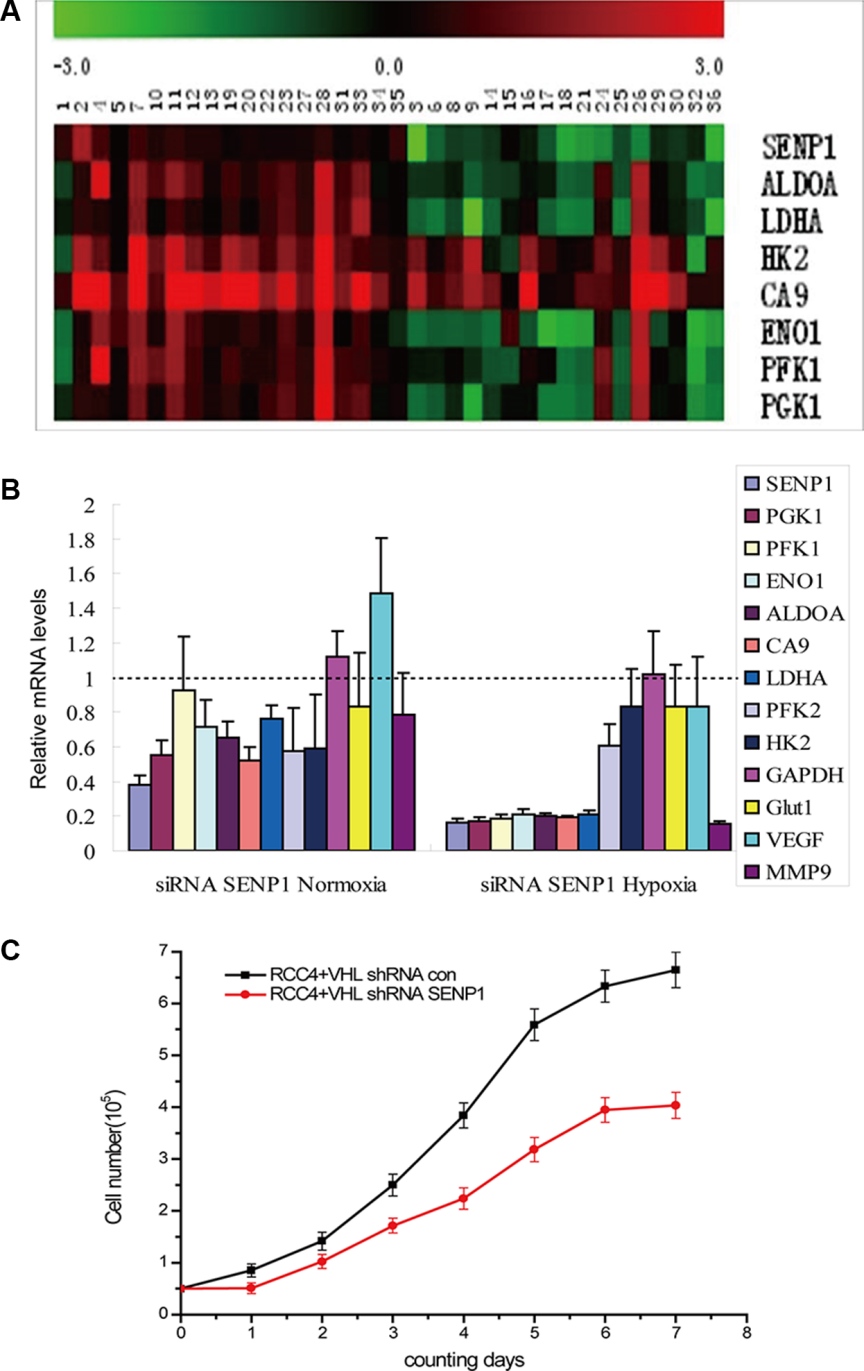
SENP1 upregulates the expression of key glycolytic enzymes and inhibits cell proliferation in ccRCC (**A**) Heat map showed correlation between the mRNA levels of SENP1 and key glycolytic enzymes in ccRCC tissues. mRNA levels of tumors were normalized against the corresponding mRNA levels of the paired adjacent tissues. Heat map scale bar indicates log 10-fold change in the mRNA levels of the indicated genes. Red: High expression; Green: Low expression; Black: no change. (**B**) The effect of SENP1 on the expression levels of glycolytic enzymes under normoxic and hypoxic conditions. The mRNA levels of SENP1 and hypoxia response genes (including *PGK1*, *PFK1*, *ENO1*, *ALDOA*, *CA9*, *LDHA*, *PFK2*,*HK2*, *GAPDH*, *GLUT-1*, *VFGF* and *MMP-9*) in SENP1 knockdown RCC4/VHL cells and the non-specific target siRNA transfected control RCC4/VHL cells were determined by qRT-PCR, before and after treatment of hypoxia. Expression of glycolytic enzymes are frequently suppressed in SENP1 knockdown RCC4/VHL cells under normoxic and hypoxic condition. The data presented are corrected by the non-specific target siRNA transfected control cells. The data represent the mean of three separate experiments ± the SD. (**C**) The effect of SENP1 on the growth of ccRCC cells. Comparison of proliferation curves between RCC4/VHL cells with and without SENP1 knockdown. The experiment was performed in triplicate, the data represent the mean of four replicate samples ± the SD from one representative experiment.

To identify the effect of SENP1 on glycolysis in ccRCC, the mRNA expression levels of the key glycolytic enzymes, including *PGK1*, *PFK1*, *ENO1*, *ALDOA*, *CA9*, *LDHA,* and *HK2,* in ccRCC tumor and adjacent normal tissues were examined by real-time RT-PCR. Correlation between SENP1 expression and the levels of these key enzymes are shown using a heat map (Figure 2A); with the exception of *CA9* and *HK2*, expression levels of most tested enzymes are positively correlated with SENP1 expression level.

Next, we explored whether SENP1 could directly affect glycolysis in ccRCC. We tested the expression levels of the above key glycolytic enzymes and two hypoxia response genes, *VFGF* and *MMP-9*, after SENP1 was stably knocked down in the RCC4/VHL cell line. As expected, SENP1 knockdown noticeably decreased the expression levels of the majority of those hypoxia response genes, compared to control RCC4/VHL cells transfected with non-specific target shRNA. In addition, under hypoxic condition, a greater reduction could be found in the expression levels of *PGK1*, *PFK1*, *ENO1*, *ALDOA*, *CA9*, *LDHA,* and *MMP9* in SENP1 knockdown cells (Figure 2B). These results indicate that SENP1 is a positive upstream regulator of the hypoxia-induced expression of key glycolytic enzymes in ccRCC, which in turn promote glycolysis.

It is well known that hypoxia occurs frequently in human cancers as a result of rapid cell proliferation and insufficient blood supply [27]. To adapt to the hypoxic circumstance, the protein levels of hypoxia-inducible factors (HIFs) increase, and induce expression of downstream genes including glycolytic enzymes. The resulting enhanced glycolytic flux provides building materials for formation of cell structure and energy for survival or proliferation of tumor cells. Consistent with our speculation, knockdown of SENP1 in RCC4/VHL cells significantly reduced cell proliferation under hypoxic conditions (Figure 2C). Taken together, the above observations implied that SENP1 promotes ccRCC proliferation by increasing glycolysis under the condition of hypoxia.

### SENP1 upregulates the expression of glycolytic enzymes through HIF-1α deSUMOylation and stabilization

**Figure 3:**
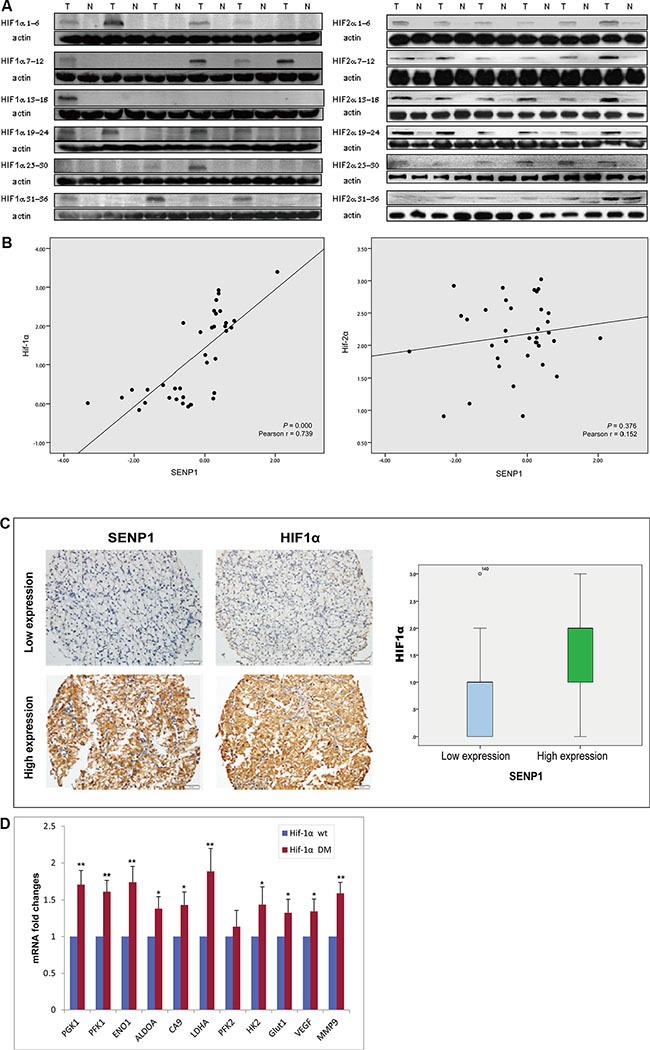
SENP1 upregulates the expression of glycolytic enzymes through HIF-1α deSUMOylation and stabilization (**A**) HIF-1α and HIF-2α were overexpressed in ccRCC tissues. Expression of HIF-1α and HIF-2α was determined by western blotting on 36 matched pairs of ccRCC tumor and adjacent normal tissues. (**B**) The correlation between SENP1 expression and HIF-1α or HIF-2α expression in ccRCC tissues was evaluated by Pearson correlation analysis. X axis indicates log 10 fold change in SENP1 mRNA level, Y axis indicates log 2 fold change in the protein level of HIF-1α or HIF-2α. (**C**) Expression of SENP1 and HIF-1α was determined by immunohistochemistry on a ccRCC tissue microarray (TMA) consisting of 145 samples. Box-plots indicate the expression level of HIF-1α in SENP1 high- and low-expression groups. (**D**) Expression levels of downstream metabolic genes of HIF-1α (including *PGK1*, *PFK1*, *ENO1*, *ALDOA*, *CA9*, *LDHA* and *HK2*) were detected by qRT-PCR, after wildtype (wt) or SUMOylation site mutant (DM) HIF-1α was transfected into SENP1 stable knockdown RCC4/VHL cells. Y axis shows the fold changes in the average mRNA levels of the indicated genes in HIF-1α (DM) transfected cells compared with HIF-1α (wt) transfected cells. The data represent the mean of three independent experiments ± the SD. Statistical significance was analyzed by Student's *t-test*, *P < 0.05, **P < 0.01.

HIF-1α/2α are the key regulators of the tumor cell response to hypoxia. In previous work, using *SENP*^−/−^ MEF cells, we uncovered the critical role of SENP1 in regulating stabilization of HIF-1α by removing SUMO from SUMO-modified HIF-1α, which enhanced transcriptional activation of HIF-1α [14]. Since SENP1 upregulates glycolysis under hypoxic conditions, we asked whether this process is mediated by SENP1 regulation of HIF-1α. We measured the protein levels of HIF-1α and HIF-2α in the 36 matched pairs of ccRCC tumor and adjacent normal tissues by immunoblotting. As shown in Figure 3A, both HIF-1α and HIF-2α are more highly expressed in ccRCC tumor tissues than in paired adjacent normal tissues. Using scatter plot, we found a strong positive correlation between expression levels of SENP1 and HIF-1α (*P* < 0.01), but no correlation between SENP1 and HIF-2α expression levels (Figure 3B). This observation was further confirmed by the detection of SENP1 and HIF-1α/2α using immunohistochemistry in a tissue microarray (TMA) of 145 human ccRCC samples (Figure 3C, *P* < 0.01).

To further demonstrate the involvement of the SENP1/HIF-1α axis in the regulation of glycolysis, we transfected wildtype or SUMOylation site mutant HIF-1α into SENP1 stable knockdown RCC4/VHL cells, and then, measured the expression levels of some downstream genes of HIF-1α, which encode glycolytic enzymes, including *PGK1*, *PFK1*, *ENO1*, *ALDOA*, *CA9*, *LDHA,* and *HK2*. As shown in Figure 3D, the mRNA levels of the metabolic genes were elevated in HIF-1α DM (SUMOylation site mutant) transfected cells compared to wildtype HIF-1α, indicating deSUMOylation of HIF-1α is a crucial step in regulating glycolysis by SENP1.

### Expression level of SENP1 is correlated with poor clinical outcome of ccRCC

**Figure 4:**
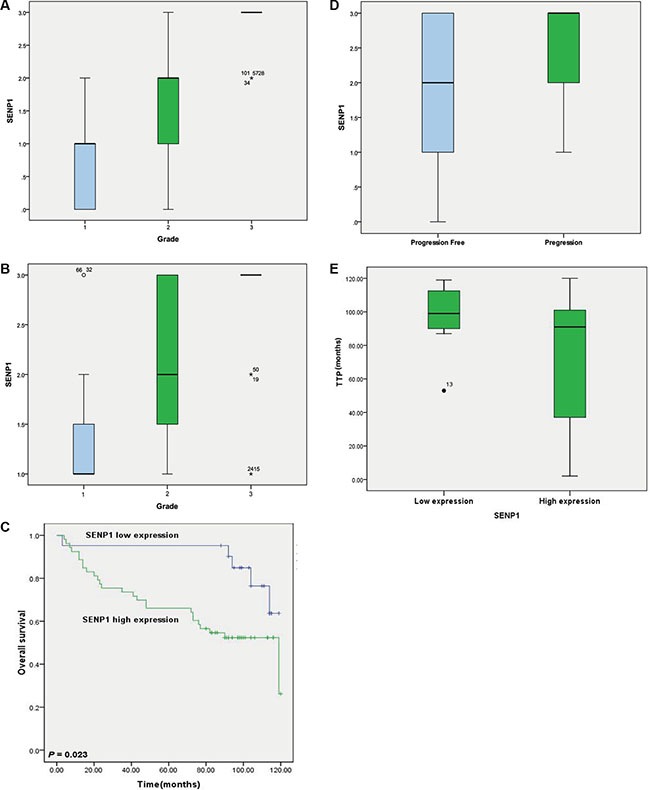
Expression level of SENP1 is correlated with poor clinical outcome of ccRCC (**A**) The association of the protein level of SENP1 with grade of ccRCC tumor tissues was analyzed in the tissue microarray (TMA) section containing 145 ccRCC specimens using one-way ANOVA followed by Tukey's test, *P* < 0.01. (**B**) Correlation of SENP1 expression with grade of ccRCC was validated in another group of ccRCC tumor tissues which obtained from 74 patients with long term follow-up data. Statistical significance was analyzed by one-way ANOVA with Tukey's test, *P* < 0.01. (**C**) Kaplan-Meier survival curve evaluated the relationship of SENP1 expression level with clinical outcome in ccRCC patients. Log-rank test was used to calculate *P value*, *P* = 0.023. (**D**) SENP1expression correlated with tumor progression in ccRCC patients with stage I-III after radical nephrectomy. Statistical significance was analyzed by Student's *t-test*. *P* = 0.039. (**E**) Time to progression (TPP) of ccRCC patients was compared between groups of high and low level of SENP1 expression. Statistical significance was analyzed by Student's *t-test*, *P* = 0.000.

To investigate the clinical significance of SENP1, we analyzed the association of SENP1 expression with clinicopathological factors in the samples contained in the TMA. We found SENP1 expression positively correlated with tumor grade (*P* < 0.01) (Figure 3C, Figure 4A); patients with higher tumor grade also showed higher SENP1 expression than those with low tumor grade. This result was further confirmed by the correlation between SENP1 expression and clinicopathological factors in another group of 74 ccRCC patients with long term follow-up data (Table 3, Figure 4B). In addition, we observed a trend showing elevated SENP1 expression in the ccRCC with advanced stages (stage III and IV, 80.0%) than those with early stages (stage I and II, 67.3%), and with tumor invasion (85.7%) than without tumor invasion (68.3%), although there is no statistical significance (Table 3).

**Table 3:** Association of SENP1 expression with clinical pathological factors in ccRCC

Variables	Patients (*n*)	Expression of SENP1		χ^2^	*P*
		Low (%)	High (%)		
**Age**					
< 60	38	14 (36.8)	24 (63.2)	2.753	0.097
≥ 60	36	7 (19.4)	29 (80.6)		
**Sex**					
Male	49	14 (28.6)	35 (71.4)	0.003	0.959
Female	25	7 (28.0)	18 (72.0)		
**Grade**					
I	16	12 (75.0)	4 (25.0)	18.413	0.000^**^
II	28	7 (25.0)	21 (75.0)		
III	30	2 (6.7)	28 (93.3)		
**TNM stage**					
I	16	7 (43.8)	9 (56.2)	3.304	0.347
II	33	9 (27.3)	24 (72.7)		
III	11	3 (27.3)	8 (72.7)		
IV	14	2 (14.3)	12 (85.7)		
**Size**					
< 5 cm	11	4 (36.4)	7 (63.6)	1.587	0.662
5–7 cm	14	5 (35.7)	9 (64.3)		
8–10 cm	24	7 (29.2)	17 (70.8)		
> 10 cm	25	5 (20.0)	20 (80.0)		
**Tumor invasion**					
Negative	60	19 (31.7)	41 (68.3)	1.687	0.194
Positive	14	2 (14.3)	12 (85.7)		
**Lymph node metastasis**					
Negative	65	19 (29.3)	46 (70.7)	0.191	0.662
Positive	9	2 (22.2)	7 (77.8)		
**Metastasis**					
Negative	69	19 (27.5)	50 (72.5)		0.618^b^
Positive	5	2 (40)	3 (60)		

** *P* < 0.01;

b Fisher's Exact Test. Statistical analysis showed significant positive association between SENP1expression and tumor grade.

Subsequently, we analyzed the association of the above clinicopathological variables or SENP1 expression with ccRCC clinical outcome. As shown in Table 4, age, tumor grade, clinical TNM stage, tumor invasion, and lymph node, and distant metastasis were significantly associated with clinical outcome. Moreover, patients with high SENP1 expression level had shorter overall survival than patients with low SENP1 expression (mean, 108.9 versus 80.7 months, *P* = 0.023, Table 4, Figure 4C). Furthermore, in ccRCC patients with clinical TNM stage I–III after radical nephrectomy, SENP1 expression correlated with tumor progression, and patients with high SENP1 expression were more likely to suffer disease progression (*P* = 0.039, Figure 4D), and markedly shorter time to disease progression (*P* < 0.001, Figure 4E). Collectively, the data suggest that high expression of SENP1 might be a prognostic indicator of worse clinical outcome in ccRCC.

**Table 4:** Univariate analysis of overall survival in ccRCC according to clinical pathological factors

Variables	Patients (*n*)	Estimated survival (mean ± SD)	*P*^a^
**Age**			
< 60	38	104.246 ± 5.109	0.002^**^
≥ 60	36	70.869 ± 7.501	
**Sex**			
Male	49	96.845 ± 7.686	0.32
Female	25	83.977 ± 6.234	
**Grade**			
I	16	103.545 ± 7.139	0.000^**^
II	28	98.735 ± 6.953	
III	30	68.263 ± 8.093	
**TNM stage**			
I	16	104.816 ± 4.852	0.000^**^
II	33	103.011 ± 5.309	
III	11	78.909 ± 12.316	
IV	14	34.071 ± 9.725	
**Tumor size**			
< 5 cm	11	106.636 ± 7.974	0.121
5–7 cm	14	78.786 ± 9.967	
8–10 cm	24	88.33 ± 8.624	
> 10 cm	25	82.04 ± 9.044	
**Tumor invasion**			
Negative	60	98.332 ± 4.597	0.000^**^
Positive	14	45.169 ± 11.454	
**Lymph node metastasis**			
Negative	65	97.065 ± 4.436	0.000^**^
Positive	9	23.889 ± 9.939	
**Metastasis**			
Negative	69	91.438 ± 4.932	0.015^*^
Positive	5	46.40 ± 18.301	
**SENP1**			
Low expression	21	108.885 ± 5.569	0.023^*^
High expression	53	80.709 ± 6.207	

* *P* < 0.05,

** *P* < 0.01.

a Kaplan-Meier method and log-rank test. Age, grade, TNM stage, tumor invasion, lymph node and distant metastasis and SENP1 expression were significantly associated with overall survival.

## DISCUSSION

As almost all of the inherited renal cancer syndromes caused by germline mutations identified to date exhibit disorders in oxygen, iron, nutrient, or energy sensing, RCC has been viewed as a metabolic disease [28]. We and others have previously demonstrated that clear cell renal-cell carcinoma (ccRCC), the most common RCC subtype, features different metabolite profiles compared to adjacent normal kidney tissues, including elevated levels of lactate, glutamate, pyruvate, glutamine, and creatine, but decreased levels of acetate, malate, and amino acids such as valine, alanine, and aspartate, indicating enhanced glycolysis and diminished tricarboxylic acid (TCA) cycle activity in ccRCC [21–24]. Increased glycolysis may allow the diversion of glycolytic intermediates into various biosynthetic pathways, including those generating nucleosides and amino acids, which in turn, facilitates the biosynthesis of macromolecules and organelles required for assembling new cells [29].

In a previously study, we reported the essential role of Sentrin/SUMO-specific protease 1 (SENP1) in modulating VHL mediated ubiquitin-proteasome degradation of HIF-1α and subsequent VEGF, Glut-1, and EPO expression, under hypoxic conditions [14, 19]. Considering HIF-1α transcriptionally activates key glycolytic enzymes, we speculated that SENP1 may also be associated with glycolysis. Here, consistent with our speculation, integrated gene expression and metabolomic analyses of 36 matched pairs of ccRCC tumor and adjacent normal tissues revealed that patients with high SENP1 expression level also exhibit enhanced glycolysis, illustrated by elevated levels of lactate and pyruvate. Interestingly, in the SENP1-high expression group, we observed elevated levels of citric acid cycle intermediates, malate and succinate, which might be explained by SENP1 regulation of mitochondrial biogenesis and function through PGC-1α [26]. TCA cycle intermediates, such as succinate, fumarate and oxaloacetate, inhibit the activity of prolyl hydroxylases (PHD), which catalyze the hydroxylation of proline residues in HIF α subunits. As the hydroxylated proline residues in HIF α subunits are recognized by pVHL and targeted for proteosomal degradation, inhibition of PHD by these TCA cycle metabolites will stabilize HIF α subunits and lead to pseudo-hypoxia in tumor, and thereafter, promote tumor formation and development [30]. In addition, metabolites like acetyl-coA and NAD are implicated in epigenetic modification acting as enzyme cofactors, whose levels are affected by the activity of TCA cycle [31]. Therefore, upregulating levels of malate and succinate may be one of the ways that SENP1 functions as an oncoprotein, a topic that needs further investigation.

Enzymes, especially rate-limiting enzymes, determine the direction, strength, and speed of metabolic pathways; correlation analysis showed increased levels of glycolytic enzymes in SENP1 high-expression tumors, including *PGK1*, *PFK1*, *ENO1*, *ALDOA*, *CA9*, *LDHA* and *HK2*. Furthermore, SENP1 knockdown in RCC4/VHL cells noticeably reduced levels of key glycolytic enzymes under both normoxic and hypoxic conditions, providing evidence for SENP1 upregulation of glycolysis in ccRCC.

SENP1 deSUMOylates target proteins, which can positively or negatively affect protein stability. In many cases, SUMO competes with ubiquitin for substrate binding, in which case, SUMOylation is believed to protect target proteins from proteosomal degradation [32–34]. However, according to our previous work and other studies, SUMO and ubiquitin conjugate to different lysine binding sites on HIF-1α. SUMOylation may act as a signal to the E3 ligase VHL prompting HIF-1α degradation. Hence, deSUMOylation of HIF-1α by SENP1 could prevent degradation of HIF-1α [14, 35, 36]. Consistent with this notion, the results presented here showed a positive correlation between SENP1 expression and HIF-1α protein levels in ccRCC tissues. Furthermore, when the SUMOylation sites of HIF-1α (K391 and K477) were mutated, overexpression of this mutant HIF-1α in SENP1-knockdown RCC4/VHL cells increased the expression levels of key glycolytic enzymes compared to wild-type HIF-1α, suggesting deSUMOylating of HIF-1α is an important step for SENP1 regulation of glycolysis. It is of note that, although levels of both HIF-1α and HIF-2α increased in tumor tissues compared to matched adjacent normal tissues, no significant correlation was observed between HIF-2α protein levels and SENP1 expression, suggesting the effect of SENP1 is specific to HIF-1α in ccRCC. However, as it has been reported that hypoxia could inactivate VHL through PIASy-mediated SUMO modification [37], it is possible that SENP1 directly regulates the SUMOylation status of VHL, which warrants further exploration.

A number of studies have shown that SENP1 functions as an oncoprotein during tumorigenesis and cancer progression. We previously revealed that SENP1 promotes prostate tumor growth and metastasis [16], and is overexpressed in most colon cancer tissues and essential for colon cancer cell growth [38]. Others reported that SENP1 affects cell survival, proliferation, and apoptosis in multiple myeloma [39], cell migration and invasion of neuroblastoma [40], and sensitivity of hypoxic ovarian cancer cell to cisplatin [41]. One report showed that SUMOylation-defective MITF germline mutation predisposes carriers to melanoma and renal carcinoma [11], but, so far, there is no report on a clear role of SENP1 in renal cell carcinoma. In this study, we found knockdown of SENP1 inhibits the proliferation of RCC4/VHL cells under hypoxic conditions, and SENP1 expression is positively associated with tumor grade in ccRCC, suggesting it as a potential biomarker for tumor differentiation and aggressiveness. In addition, we found that patients with high SENP1 expression level have shorter overall survival (OS) and time to progression (TTP) than patients with low SENP1 expression level, and are prone to advanced disease progression. These results suggest SENP1 may serve as a prognostic indicator for poor clinical outcome in ccRCC.

VHL is biallelically inactivated via point mutation, deletion, or methylation in most sporadic ccRCC [42–44]. The reported incidence of somatic VHL mutations in sporadic ccRCC varies up to 91% [45, 46]. However, some mutant VHL variants do not affect stabilization of HIF α [47]. Previous studies suggest that inactivation of the VHL gene is an early step in the development of ccRCC [48–52], that most VHL-negative ccRCCs never metastasize [53], and that VHL mutation status does not provide useful prognostic information for patients with ccRCC as yet [47, 55–57]. The frequency of VHL gene mutation was only 35% in the tissues profiled in this study. Hence, our data provide a possible mechanism, apart from inactivity of VHL, for tumorigenesis and progression of ccRCC.

To summarize, we reported here for the first time that SENP1 promotes cell proliferation and disease progression in ccRCC, possibly through deSUMOylating and stabilizing HIF-1α, leading to increased expression of key glycolytic enzymes and enhanced glycolytic flux. Moreover, SENP1 expression level may provide a prognostic indicator of poor clinical outcome in ccRCC.

## MATERIALS AND METHODS

### Patients and tissue specimens

36 pairs of freshly frozen clear cell renal cell cancer (ccRCC) and adjacent normal tissue samples, and 74 formalin-fixed and paraffin-embedded ccRCC tissue specimens were obtained from the Kidney Cancer Tissue Bank in Renji Hospital, Shanghai Jiao Tong University School of Medicine (Shanghai, China). Histopathology of the specimens was determined by pathologists in the hospital, and patient identifiers were removed before analysis. Samples were catalogued, and clinical information including survival data on cases, was obtained through the hospital's kidney cancer database. The ccRCC specimens were graded by the Fuhrman nuclear grading system and staged according to the 2002 version of Tumour Nodes Metastasis (TNM) system proposed by the International Union Against Cancer (UICC) and the American Joint Committee on Cancer (AJCC). All cases did not receive immune therapy, chemotherapy, embolism, or other anticancer therapy before nephrectomy. Informed consent was obtained from each patient before operation. The freshly frozen specimens were used for Real-time PCR, Western Blotting, and metabonomics analysis. Formalin-fixed and paraffin-embedded tissues were examined by IHC assay for further survival analysis.

Tissue microarray (TMA) slides for ccRCC were obtained from National Engineering Center for Biochip at Shanghai, the TMA cores that were difficult to classify (due to technical artifacts such as folds in the tissue, air bubbles, and cores overlapping, or due to difficulty in morphological classification) were eliminated from the analysis in order to categorize the tissue appropriately. After careful quality analysis of each core on the TMA by pathologists, 145 high quality samples (duplicate cores of each) were considered appropriate. The TMA slides were used for IHC assay to detect the expression level of SENP1and HIF-1α.

All protocols were approved by the Ethics Committee of Renji Hospital, Shanghai Jiao Tong University School of Medicine. Informed consent was obtained from all patients before study inclusion.

### Cell culture and transfection

RCC4/VHL cells (purchased from ECACC) were cultured in DMEM with 10% FBS (Gemini). Hypoxia treatment (2% O_2_) was performed in a specially designed hypoxia incubator (Thermo Electron, Forma, MA, USA) and generated by flushing a mixture of air and N_2_ in combination with 5% CO_2_.

21- nucleotide siRNAs targeting SENP1 (5′-AACTACATCTTCGTGTACCTC) were synthesized (Dharmacon). The above siRNA in an inverted orientation was used as a non-specific siRNA control. The SENP1 and non-specific siRNA oligos were inserted into a pSuppressorNeo vector (IMGENEX Corporation) according to manufacturer's instructions. RCC4/VHL cells were transfected with the siRNA plasmids using lipofectamine 2000 (Invitrogen) and selected by G418. Silencing efficiency of the siRNA was confirmed by performing real-time PCR to examine SENP1 expression. To explore the function of HIF-1α in SENP1 mediated glycolysis, wild type or SUMO dysfunctional HIF-1α mutant (K391R and K477R) was transfected into the stable SENP1 knockdown RCC4/VHL cells with lipofectamine 2000.

### ^1^H NMR based metabolic changes assay in tissues

The ^1^H NMR experiments and PLS-DA analysis were performed as described previously [21, 22]. The concentrations of target metabolites were quantified from the spectra using trimethylsilyl-propionic-2,2,3,3d_4_-acid (TSP) as the internal reference and normalized to the weight of the tissues, which are shown in the unit of mmol/kg wet tissue weight.

### Real-time quantitative PCR

Total RNA was isolated from tissues or cells by Trizol reagent (Invitrogen, CA, USA). RNA was treated with DNase (Promega, Madison, WI, USA). Complementary DNA was synthesized using cDNA synthesis kit (Takara, Shiga, Japan) according to the manufacturer's instructions. Fluorescence real-time RT-PCR was performed with the double-stranded DNA dye SYBRGreen PCR Core Reagents (PE Biosystems, Warrington, UK) using the ABI PRISM 7300 System (PerkinElmer, Torrance, CA, USA). All data were analyzed using ABI PRISM SDS 2.0 software (PerkinElmer). PCR was carried out in triplicate and standard deviations representing experimental errors were calculated. To study the correlation between mRNA expression levels of targeted genes, we converted the relative mRNA expression values applying log 10 and performed heat map analysis using Java Tree View 35. Pairs of PCR primers used to amplify the target genes are shown below: SENP1(Forward: 5′-ACGACTCCATGGGTGGGATAA

ACA, Reverse: 5′-TTTGCAGGCAAACATCCCACAG

TC; PFKL(Forward: 5′-TGGGAGCTTCGAGAACAA

CTGGAA, Reverse: 5′-ATTCAGGATGGCCAGGGAGA

AGTT); ALDOA(Forward: 5′-GGCCATGCTTGCACT

CAGAAGTTT, Reverse: 5′-AATGGCATTGAGGTTGAT

GGACGC); ENO1(Forward: 5′-TACCTTCATCGCTGAC

CTGGTTGT, Reverse: 5′-TGCCCAGCTCCTCTTCAAT

TCTGA; GAPDH (Forward: 5′-CATGTTCGTCATGG

GTGTGAACCA, Reverse: 5′-AGTGATGGCATGGACT

GTGGTCAT); HK2(Forward: 5′-TGAAGTTGGCCTCA

TTGTTGGCAC, Reverse: 5′-TTCTCCTTCCCCAGTT

CCACGTT); LDHA(Forward: 5′-GTGCACCCAGTTTC

CACCATGATT, Reverse: 5′-TTCTTCAAACGGGCCTC

TTCCTCA); PGK1(Forward: 5′-TGGACAAGCTGGAC

GTTAAAGGGA, Reverse: 5′-AATTTGATGCTTGGGAC

AGCAGCC); 18S (Forward: 5′-AGGCCCTGTAATTG

GAATGAGTC, Reverse: 5′-GCTCCCAAGATCCAAC

TACGAG).

### Western blot analysis

Tissues were lysed in RIPA buffer (20 mM Tris pH 7.5, 150 mM sodium chloride, 1% Nonidet P-40, 0.5% sodium deoxycholate, 1 mM EDTA, 0.1% SDS) containing complete mini protease inhibitors (Roche) and phosphatase inhibitors. Western blots were performed using 20~50 μg of lysate protein, and membranes were incubated with antibodies against HIF-1α (1:500; NB 100-105, Novus, CO, USA) or HIF-2α (1:1000; NB 100-132, Novus, CO, USA).

### Immunohistochemistry

Immunohistochemistry was performed on 5-μm sections of the tissues to assess expression of SENP1 and HIF-1α using the standard streptavidin-biotin immunoperoxidase method. The sections were deparaffinized in xylene, rehydrated in alcohol gradient, and rinsed in distilled water. Endogenous peroxidase activity was blocked by incubation in 3% hydrogen peroxide-methanol for 10 min. After washing with PBS, the slides were blocked with 10% goat serum in PBS at room temperature for 1 hour, and then incubated with antibody against SENP1 (1:50; ab58417, Abcam, UK) or HIF-1α (1:200; ab463, Abcam, UK) overnight at 4°C. The EnVision^+ TM^ (Dako, Carpinteria, CA, USA) was then added to the sections and incubated for 1 hour. Finally, avidin-biotin-peroxidase reagents were added, and the resulting peroxidase activity was revealed by incubating the slides with 3,3- diaminobenzidine for 10 min, followed by haematoxylin counterstaining. Negative control sections were performed by using PBS instead of the specific primary antibody.

All samples were reviewed by two pathologists to assign scores. The average score for duplicate cores from each sample was calculated, intensity scores of 0, 1, 2, and 3 represented negative, weak, moderate, or strong staining, respectively. Distribution scores of 0,1,2,3, and 4 were assigned for < 10%, 10%–30%, > 30%–50%, > 50%–80%, or > 80% positive cells, respectively. Sum scores could be obtained according to both the intensity scores and the distribution scores, which was used to divide the samples into 4 groups as follows: (0) negative, 0–1; (1) weak, 2–3; (2) moderate, 4–5; and (3) strong, 6–7. (0) and (1) were considered to be low expression, (2) and (3) were considered to be high expression.

### Cell proliferation assays

RCC4/VHL cells with or without SENP1 knockdown were plated into 12 well plates (5 × 10^4^ cells/well) in DMEM medium supplemented with 10% FBS, the medium was changed every two days. The numbers of viable cells per well were counted at each time point.

### Statistical analysis

Statistical analysis was carried out using SPSS 19.0 software for windows (SPSS Inc, Chicago, IL, USA). For measurement data, Student's *t-test* was used to determine the differences between two means, and one-way ANOVA followed by Tukey's test were employed to compare three or more means. For enumeration data, Chi-square test or Fisher's Exact Test was used for analysis of differences. Survival curves were analyzed by the Kaplan-Meier method and compared with the log-rank test. Statistical significance was determined when a *P value* is less than 0.05.
